# Clinical spectrum of tuberculosis among HIV infected patients in India: Correlation with immunological status using CD4 counts

**DOI:** 10.6026/973206300211602

**Published:** 2025-06-30

**Authors:** Snigdha M.K., Krishnavajhala Padma Theja, Kondeti Ganga Bhavani, Chennakesavulu Dara, Pranavi Paloju, Shiva Kumar Nimma

**Affiliations:** 1 Department of General Medicine, ESIC Medical College, Sanathnagar, Hyderabad-500038, India

**Keywords:** Tuberculosis, HIV, CD4 count, pulmonary TB, extra pulmonary TB, immunological status, opportunistic infections

## Abstract

Tuberculosis (TB) remains a common and serious opportunistic infection among HIV-infected individuals, with clinical presentation closely tied to immune status. In this prospective study of 100 HIV-positive patients with TB, pulmonary TB predominated in those with CD4 counts >200, while extra pulmonary and disseminated TB were strongly associated with CD4 counts <200. Symptoms such as cough and fever were prevalent and radiographic findings varied with immune suppression levels. CD4 counts served as a critical marker in determining the clinical spectrum and severity of TB. These findings underscore the importance of CD4-based assessment in guiding timely diagnosis and targeted management of TB in HIV patients.

## Background:

Tuberculosis (TB) continues to be an important public health issue in resource-poor nations such as India, where TB and HIV co-exist very frequently [1]. The most potent recognized risk factor for latent to active TB progression is HIV infection, increasing the lifetime risk from 10% among immunocompetent persons to more than 60% in those who are co-infected. India, where the global burden of TB lies close to a quarter, is confronted by an added burden caused by the growing tide of HIV infections [2]. TB is the single most frequent opportunistic infection in HIV-infected persons and is usually due to reactivation of latent infection, although primary infection risk is also greatly heightened [3]. Clinical manifestation of TB among HIV-infected individuals is immune status-dependent; those with a good CD4 count present with classical pulmonary TB, whereas in advanced immunosuppression, atypical or extrapulmonary disease is more common [4]. Depressed CD4 count is related to characteristics such as disseminated TB, lack of cavitations and diagnostic complexity. Due to the overlap in symptoms with other opportunistic infections, correct diagnosis is usually based on thorough clinical and laboratory assessment [5]. It is important to know these associations to ensure early diagnosis and treatment of TB among HIV-infected patients, especially in resource-poor settings.

## Materials and Methods:

This potential observational investigation was undertaken in 18 months, November 2018-April 2020, in Osmania General Hospital under coordination with TB Chest Hospital and ART Clinic. Consecutive samples consisting of 100 HIV-positive individuals were sampled on convenience basis. The study patients included both sexes over 12 years old, with the diagnosis of HIV by Rapid Test or ELISA and having clinical and investigative features of either pulmonary or extrapulmonary tuberculosis. Irrespective of whether or not they received antiretroviral therapy, patients were recruited. Exclusion criteria were patients under 12 years of age, those with a history of treated or defaulter TB and patients with other established causes of immunosuppression like diabetes, malignancies, hematological disorders, malnutrition, or patients on immunosuppressive therapy. Following informed consent, the study participants who met the inclusion criteria were enrolled in the outpatient department. Data was collected on pre-tested structured proforma through patient interview, complete history-taking and thorough physical examination. Baseline investigations done for all the patients were complete blood count, ESR, liver function test, renal function test, sputum smear for acid-fast bacilli (three samples), chest X-ray (posteroanterior view) and related biochemical and bacteriological investigations according to clinical presentation. Fine-needle aspiration cytology (FNAC) or biopsy of palpable peripheral lymph nodes was done and the tissue was then subjected to histopathological study and Ziehl-Neelsen staining. CD4 counts were measured using flow cytometry on the Becton Dickinson FAC Scan system. Statistical analysis was conducted with SPSS version 28. Data entry was done on Microsoft Excel and descriptive statistics were employed for summarizing the baseline characteristics. Continuous variables were represented as mean ± standard deviation or median with interquartile range depending on whether the Shapiro-Wilk normality test gave significant results. Categorical data were reported as frequencies and proportions. The chi-square test was employed to test associations of qualitative variables. Data were also graphically represented using bar charts and pie charts where necessary.

## Results:

Table 1 (see PDF) interprets that the study population was predominantly male, with the highest number of cases (46%) in the 30-39 year age group and a mean age of 33.66 ± 8.46 years. Table 2 (see PDF) interprets that fever (78%) and cough (75%) were the most frequent presenting symptoms, followed by breathlessness (62%), weight loss (56%) and diarrhoea (50%), suggesting systemic involvement in HIV-TB co-infection. Table 3 (see PDF) interprets that pulmonary TB was the most common clinical form (51%), followed by extrapulmonary TB (43%) and disseminated TB (6%). Table 4 (see PDF) interprets that extrapulmonary and disseminated TB were more frequent among patients with CD4 counts <200, while sputum-positive PTB was predominantly seen in those with CD4 >200, indicating a significant association between immune suppression and disease pattern. Table 5 (see PDF) interprets that upper zone lesions were exclusively seen in patients with CD4 >200, while mid/lower zone lesions were predominantly observed in those with CD4 <200, suggesting typical radiological patterns vary with immune status. The tables herein identify significant clinical and radiological correlations in patients with HIV-TB co-infection. The predominance in males (76%) and greatest number of cases (46%) in the 30-39 years age group is reflected in Table 1 (see PDF). Table 2 (see PDF) outlines symptoms on presentation as fever (78%) and cough (75%) were most prominent, followed by breathlessness, weight loss and gastrointestinal disturbance. Table 3 (see PDF) illustrates that the most common presentation was pulmonary TB (51%), followed by extrapulmonary TB (43%) and disseminated TB (6%). Table 4 (see PDF) shows a high association between type of TB and immune status-sputum-positive pulmonary TB correlated with CD4 >200, whereas extrapulmonary and disseminated TB were more common in patients with CD4 <200. Table 5 (see PDF) indicates that upper zone lesions occurred exclusively in CD4 >200 patients and were highly correlated with sputum positivity, whereas mid/lower zone lesions were characteristic of CD4 <200 patients, demonstrating that radiographic patterns are highly correlated with the extent of immunosuppression.

## Discussion:

This research points to the clinical and radiological range of tuberculosis in HIV-infected patients, underlining the role of immune status [6]. Male predominance (76%) and clustering of cases among the 30-39 years age group are in accordance with national figures and other studies and reflect the disease burden in the sexually active age group. The symptomatology prevalent in fever, cough and diarrhoea concurs with previous reports, but diarrhoea was higher in this group, potentially secondary to lower mean CD4 levels and higher immunosuppression [7]. The majority of patients presented with pulmonary TB (51%), followed by extra pulmonary (43%) and disseminated TB (6%), with the latter two presentations correlating with CD4 levels <200, consistent with other Indian reports [8]. The comparatively low rate of sputum positivity may be due to the fact that no culture was tested and more patients had non-cavitatory or lower zone lesions [9]. On radiology, infiltrative rather than fibrocavitatory lesions were found, with upper zone lesions being mostly noted in CD4 >200 patients and mid/lower zone lesions in patients with lower CD4 levels results that were statistically significant and in accordance with existing literature [10]. The median CD4 count was 133.78 cells/µL and the counts were significantly lower in patients with disseminated TB and sputum-negative presentations, confirming the role of immune suppression in modifying TB presentation [11]. Tuberculosis is one of the commonest opportunistic infection and chances of developing tuberculosis increases with decrease CD4 cell count [12]. These results confirm the relevance of CD4-based stratification for early diagnosis and individualized management of TB in HIV-infected patients [13]. Yet, the single-centre design of the study and small sample size are significant drawbacks, calling for larger multicentric studies for wider generalizability.

## Conclusion:

With a significant percentage of extra pulmonary and sputum-negative cases linked to CD4 counts < 200 cells/µL, pulmonary TB was shown to be the most prevalent type among HIV-infected individuals in this investigation. These results highlight the necessity of increased clinical suspicion and customised diagnostic strategies, especially in immunocompromised patients exhibiting abnormal radiographic patterns.
